# TRPV4 antagonists ameliorate ventriculomegaly in a rat model of hydrocephalus

**DOI:** 10.1172/jci.insight.137646

**Published:** 2020-09-17

**Authors:** Alexandra E. Hochstetler, Hillary M. Smith, Daniel C. Preston, Makenna M. Reed, Paul R. Territo, Joon W. Shim, Daniel Fulkerson, Bonnie L. Blazer-Yost

**Affiliations:** 1 Department of Biology, Indiana University, Purdue University, Indianapolis, Indiana, USA.; 2 Department of Radiology and Imaging Sciences, Indiana University School of Medicine, Indianapolis, Indiana, USA.; 3 Beacon Children’s Hospital, South Bend, Indiana, USA.

**Keywords:** Neuroscience, Therapeutics, Epithelial transport of ions and water, Ion channels, Neurological disorders

## Abstract

Hydrocephalus is a serious condition that impacts patients of all ages. The standards of care are surgical options to divert, or inhibit production of, cerebrospinal fluid; to date, there are no effective pharmaceutical treatments, to our knowledge. The causes vary widely, but one commonality of this condition is aberrations in salt and fluid balance. We have used a genetic model of hydrocephalus to show that ventriculomegaly can be alleviated by inhibition of the transient receptor potential vanilloid 4, a channel that is activated by changes in osmotic balance, temperature, pressure and inflammatory mediators. The TRPV4 antagonists do not appear to have adverse effects on the overall health of the WT or hydrocephalic animals.

## Abstract 2

Two distinct TRPV4 antagonists ameliorate ventriculomegaly in a genetic rat model of severe postnatal hydrocephalus, with no apparent adverse effects on animals.

## Graphical abstract

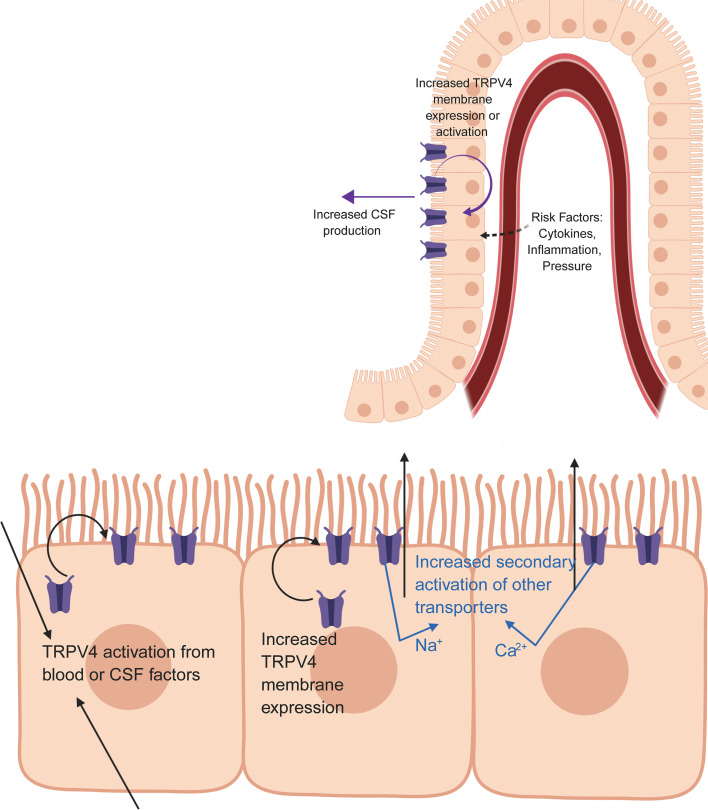

## Introduction

Hydrocephalus is a condition that affects nearly 1 million Americans and is characterized by the excessive accumulation of cerebrospinal fluid (CSF) in the brain. While people of any age may be affected, hydrocephalus is most commonly diagnosed in children. Approximately 1 in 1000 newborns will require treatment resulting in approximately 39,900 annual hospital admissions, with a cost of $2 billion dollars in the United States alone (1). Globally, hydrocephalus disproportionally affects developing nations, and there are an estimated 400,000 new patients diagnosed worldwide per year (2). Untreated hydrocephalus may cause developmental delay, visual loss, brain damage, and death.

Hydrocephalus has multiple causes resulting in abnormalities in CSF production, circulation, or absorption. In children, hydrocephalus may be congenital or associated with spinal dysraphism, premature birth, tumors, infection, hemorrhage, or trauma. Adults may develop hydrocephalus from similar causes. Teenagers and younger adults may develop idiopathic intracranial hypertension (IIH), previously called pseudotumor cerebri. This poorly understood disease is most common in young women who are overweight and may cause blindness if improperly treated. There is a growing recognition of the role of normal pressure hydrocephalus (NPH) in elderly patients. This underdiagnosed entity affects approximately 700,000 Americans per year and is one of the few potentially treatable causes of dementia (3). Posttraumatic hydrocephalus (PTH) occurs as the result of traumatic brain injury (TBI) (4–6). The reported prevalence of PTH varies considerably, with some estimates suggesting that 0.7%–29% of TBI patients have PTH (4, 5) while 45% of severe TBI patients have this condition (6).

There is no reliable pharmaceutical treatment for hydrocephalus. The only current durable treatment is surgery. Certain patients with obstructive hydrocephalus are candidates for an endoscopic procedure that creates a bypass for CSF flow called an endoscopic third ventriculostomy (ETV). Endoscopic procedures are also used to coagulate the choroid plexus (CP), leading to irreversible loss of the tissue. The long-term consequences of coagulation of the CP are unknown. The most common surgery is placement of a mechanical CSF shunt. A shunt is composed of a catheter inserted into a cerebral ventricle, a 1-way valve, and a distal catheter that drains fluid into another part of the body. Approximately 4500 new shunts are placed yearly in American children (1). Shunts have a high complication rate both in the initial surgery and in the long-term function. Approximately 8% of shunts will become infected, which leads to multiple surgeries, prolonged hospitalizations, and declines in intelligence quotient (IQ) in children. Overall, approximately 50% of all shunts in children will fail and require a revision surgery within 2 years of placement (7).

The majority of the CSF is produced by the CP, a small structure within the cerebral ventricles. The CP is composed of a fenestrated capillary network surrounded by an epithelial monolayer forming one of the most secretory epithelial tissues in the body, producing approximately 500 mL of CSF per day in an adult human. The CSF production is controlled by specific electrolyte and water channels and transporters found in the barrier epithelial cells (8). While many of the CP transporters are known, their composite activations resulting in both electrolyte and fluid flux across the epithelial cells remains incompletely characterized. The identity of the intracellular regulatory proteins and processes that control these important transport proteins are even less well defined. This is particularly true for signaling pathways and transporters that are activated by pathophysiological mechanisms such as those in play during the development of hydrocephalus.

We are studying a potential hub protein, the transient receptor potential vanilloid 4 (TRPV4) channel, which is expressed in both native CP (9) and in a CP-derived cell line (10, 11). Importantly, when TRPV4 is activated, it allows Ca^2+^ and Na^+^ to enter the cell, resulting in compensatory changes in both electrolyte and fluid movement across the epithelial monolayer. The channel can be activated by changes in osmotic balance, temperature, mechanical stress (pressure), and inflammatory mediators such as arachidonic acid metabolites (12–15), making it a hub protein that can integrate multiple stimuli. We have previously shown that, in a porcine CP epithelial cell line, stimulation of endogenous TRPV4 results in multiphasic ion fluxes, which can be blocked by either of 2 specific TRPV4 antagonists (10).

Our current studies use a rodent model of communicating hydrocephalus, the Wpk rat (*Tmem67^–/–^*) that is orthologous to a human genetic disease called Meckel-Gruber syndrome type 3 (16, 17). The affected animals carry a single nucleotide polymorphism in *Tmem67*, encoding one of a complex of proteins involved in formation of the primary cilium. The homozygous affected animals have severe hydrocephalus and renal cystic disease, and they typically survive for 18–21 days after birth (17). The *Tmem67^+/–^* heterozygous animals have milder, slowly progressing hydrocephalus, no cystic disease, and no overt symptoms of pain or distress until after the first year of life (18). In the current studies, we show that — in the homozygous, hydrocephalic animals — treatment with TRPV4 antagonists alleviates the development of ventriculomegaly. The drugs do not appear to have overt effects on the overall health of the WT or hydrocephalic animals. The effect does not appear to be the result of changes in TRPV4 synthesis and likely involves the regulation of transporter localization and activation.

Hydrocephalus confers lifelong morbidity and possible mortality to a significant number of patients. The mortality rate in patients with shunts is approximately 1% per year. Patients often suffer cognitive and emotional deficits, lower IQ, sensory deficits, depression, pain, and poor social function (19). Surgery is effective but has an inherently high complication rate and may be prohibitively expensive in developing nations. A durable, effective medical treatment may potentially revolutionize care for a large number of patients. Our goal is to provide a preclinical basis for consideration of TRPV4 as a potential drug target in the treatment of hydrocephalus, and the efficacy of TRPV4 antagonists in a genetic model of the disease represents an initial step toward that goal.

## Results

### Tmem67^–/–^ rats develop hydrocephalus that is ameliorated by treatment with 2 different TRPV4 antagonists.

**Figure 1:**
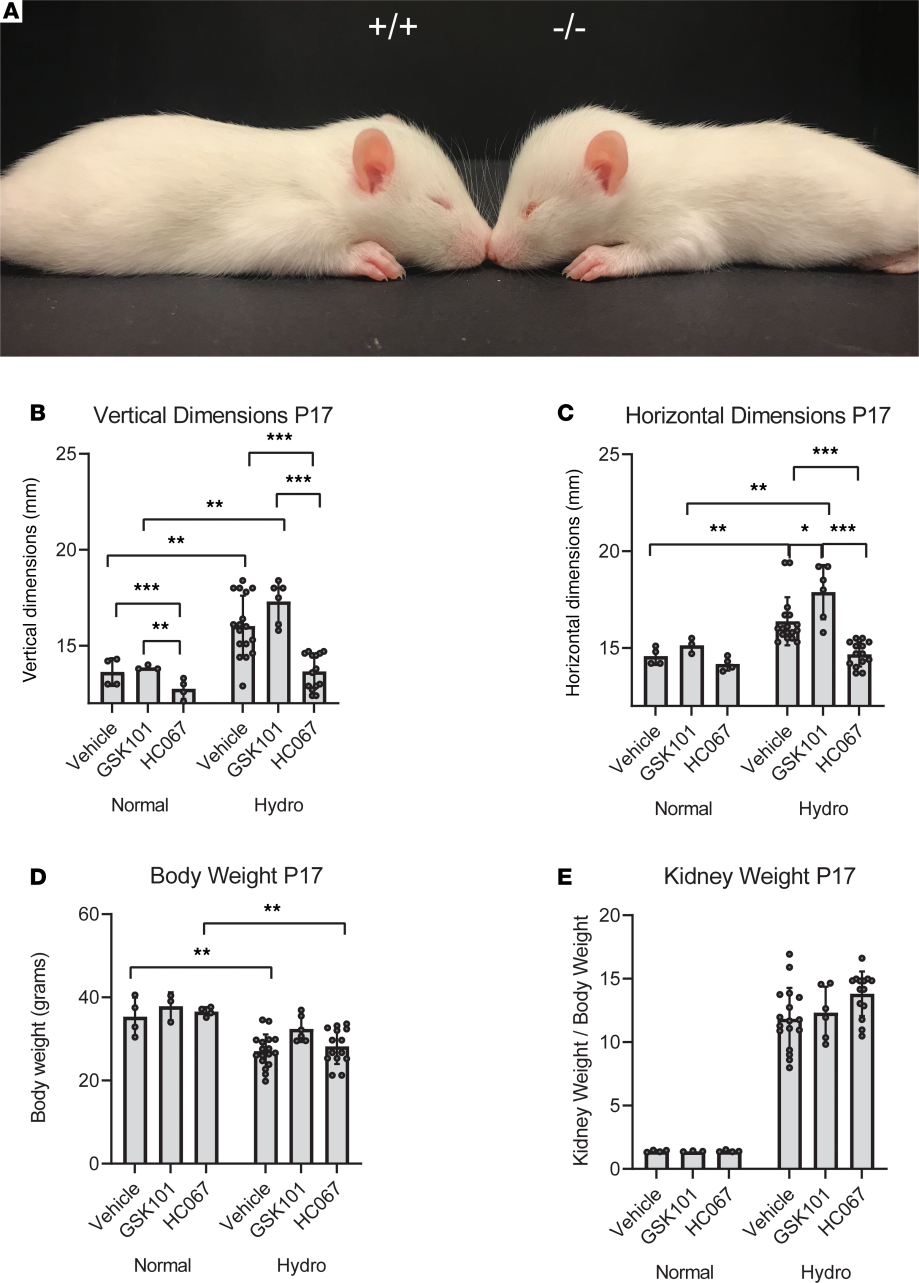
Treatment of hydrocephalic *Tmem67*^–/–^ rats with TRPV4 agonist (GSK1016790A) and antagonist (HC067047). (**A**) Images of P15 WT (*Tmem67^+/+^*) and hydrocephalic (*Tmem67*^–/–^) rats demonstrating enlarged horizontal and vertical cranial dimensions, and reduced BW compared with WT littermates. (**B–D**) Vertical and horizontal head dimensions, and BWs of normal (*Tmem67*^+/+^, *Tmem67*^+/–^) and hydrocephalic (*Tmem67*^–/–^) rats taken at P17 after 9 days of daily i.p. treatment with either vehicle, GSK101 (TRPV4 agonist), or HC067 (TRPV4 antagonist). (**E**) Kidney weights of animals, expressed as a function of BW, at P17 after 9 days of daily treatment with either vehicle, GSK101, or HC067, demonstrating no effect of the drugs on overt renal phenotype. Normal, vehicle (*n* = 4); normal, GSK101 (*n* = 3); normal, HC067 (*n* = 4). Hydro,vehicle (*n* = 17); hydro, GSK101 (*n* = 8); hydro, HC067 (*n* = 14). All data shown are the mean ± SEM for each group. Significance values were determined by 2-way ANOVA test in Prism using genotype and treatment as variables. Vehicle, DMSO/saline injection; GSK101, GSK1016790A, TRPV4 agonist, 0.003 mg/kg BW i.p. daily injection; HC067, HC067047, TRPV4 antagonist, 0.03 mg/kg BW i.p. daily injection.

**Figure 2:**
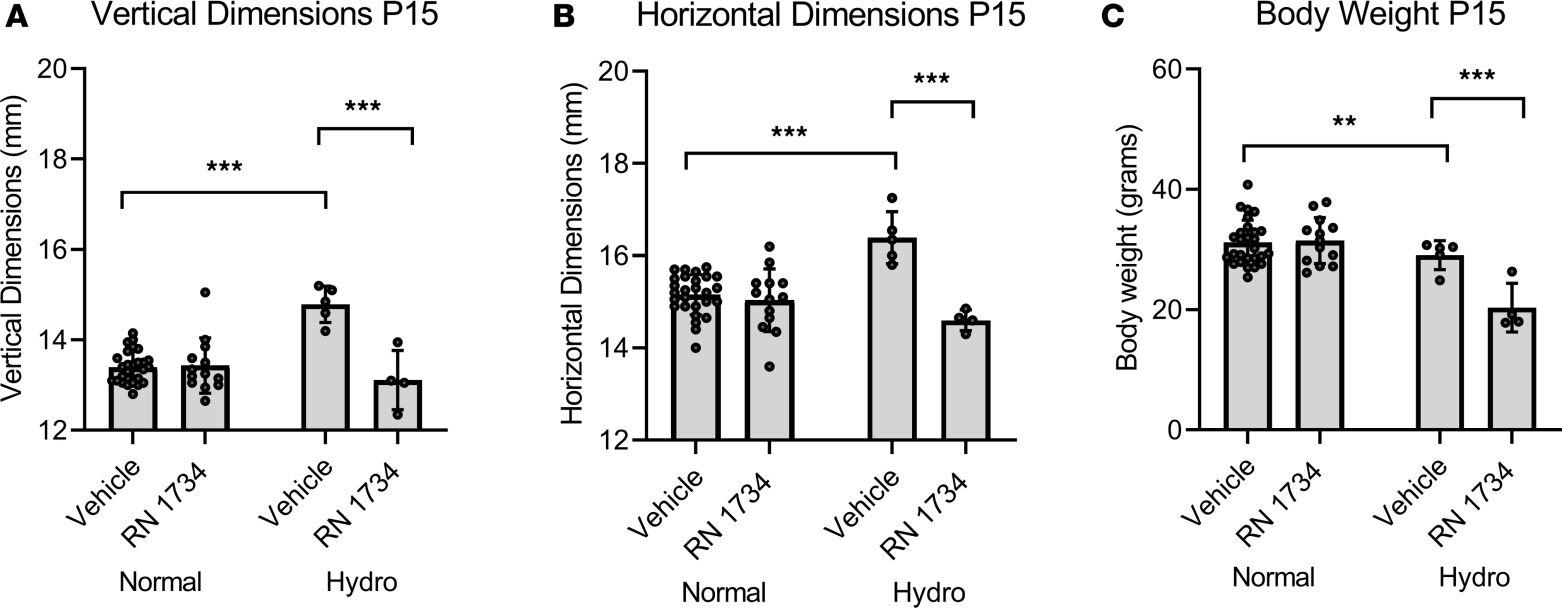
Amelioration of cranial doming by P15 in hydrocephalic *Tmem67*^–/–^ rats by treatment with TRPV4 antagonist (RN 1734). (**A** and **B**) Vertical and horizontal dimensions of normal (*Tmem67*^+/+^, *Tmem67*^+/–^) and hydrocephalic (*Tmem67*^–/–^) rats taken at P15 after 7 days daily i.p. treatment with either vehicle or RN 1734 (TRPV4 antagonist). (**C**) BWs of normal and hydrocephalic rats taken at P15 after 7 days daily i.p. treatment with either vehicle or RN 1734. Normal, vehicle (*n* = 26); normal, RN 1734 (*n* = 13). Hydro, vehicle (*n* = 5); hydro, RN 1734 (*n* = 4). All data shown are the mean ± SEM for each group. Significance values were determined by 2-way ANOVA test in Prism using genotype and treatment as variables. Vehicle, DMSO/saline injection; RN 1734, RN 1734, TRPV4 antagonist, 4 mg/kg BW i.p. daily injection.

Much like children with hydrocephalus, the *Tmem67^–/–^* rat pups develop megalocephaly (cranial enlargement and doming) (Figure 1A), a characteristic that can be used to distinguish WT and homozygous animals. We have previously shown that the hydrocephalus in this model is a communicating form of the disease (18) and has physiological effects in both the heterozygous and homozygous animals. The severity of the hydrocephalus in the heterozygous animals is not sufficient to cause doming at this early stage and can only be detected by MRI. Our initial experiments used the cranial doming to characterize the effects of 2 structurally distinct TRPV4 modulators on hydrocephalic development (Figure 1, B–E, and Figure 2, A–C).

In the first study (Figure 1), normal (WT and heterozygous) pups and hydrocephalic (homozygous) pups were treated daily for 9 days with vehicle (saline/DMSO), a TRPV4 agonist (GSK1016790A; 0.003 mg/kg BW), or a TRPV4 antagonist (HC067047; 0.03 mg/kg BW) starting on P8. Cranial dimensions were measured after 9 days of treatment. The hydrocephalic condition caused a statistically significant increase in head dimensions compared with the control animals, despite an overall decrease in BW in the affected pups. As shown in Figure 1, B and C, TRPV4 agonist treatment exacerbated the hydrocephalus in the affected animals, although this effect was only statistically significant when comparing the agonist-treated versus vehicle-treated homozygous pups in the horizontal head dimension. Conversely, treatment with the TRPV4 antagonist HC067047 ameliorated the hydrocephalus as measured by either cranial dimensions.

As a surrogate of overall health and feeding behavior, BWs were also taken at P17. Because of the disease, the BWs of the *Tmem67^–/–^* pups are consistently lower than their normal littermates. Importantly, the BWs were not further decreased by the TRPV4 antagonist treatment (Figure 1D) in the homozygous animals and treatment did not alter the BW in the normal animals. As reported previously, the *Tmem67^–/–^* pups have severe polycystic kidney disease (18). This is indicated by kidney weights as a percentage of BW (Figure 1E). The kidney weights were not significantly altered by either agonist or antagonist treatment.

As proof of principle, we conducted a similar experiment using a structurally distinct TRPV4 antagonist, RN 1734 (Figure 2). In this series, the pups were treated from P7–P14. As with the HC067047, the second antagonist significantly inhibited the cranial expansion in both dimensions. The RN 1734 antagonist was chosen for more detailed studies.

While these initial data are striking, the use of calipers to measure head dimensions is investigator dependent (experiments shown in Figure 1 and Figure 2 were conducted by a single technician who was blinded to the genotype and treatment of the animals) and is not sensitive to changes in the heterozygous animals, which were included as part of the normal cohort in the experiments shown in Figure 1 and Figure 2. We, therefore, sought to develop a more quantitative method by measuring lateral ventricular volume using MRI.

### Quantitative measurements of the effect of a TRPV4 antagonist on hydrocephalic development.

**Figure 3:**
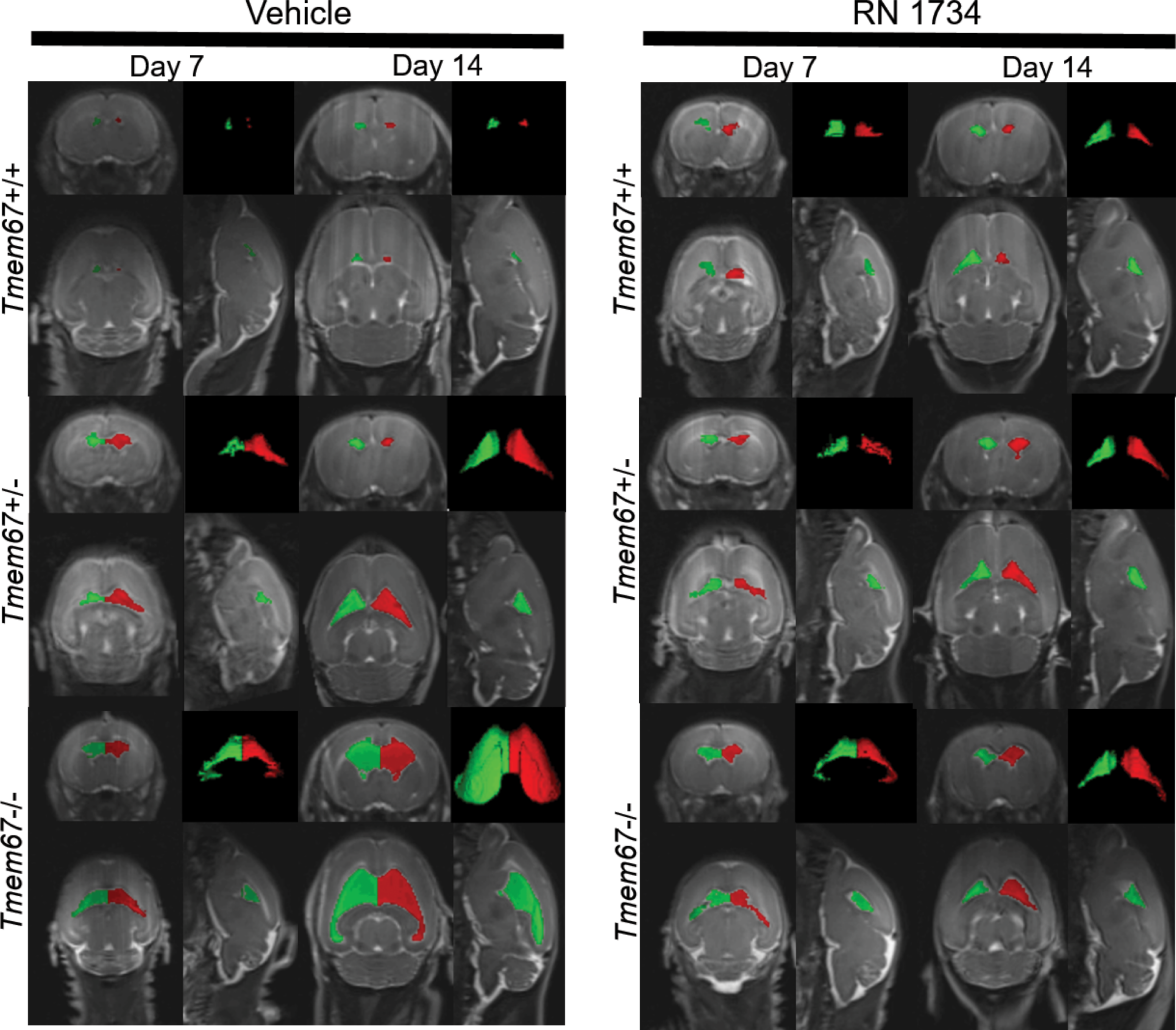
Representative MRI Scans Before and After Treatment with RN 1734. MRI Images of P7 and P14 WT (*Tmem67*^+/+^), heterozygous (*Tmem67*^+/–^), and homozygous/hydrocephalic (*Tmem67*^–/–^) rats demonstrating the size of the lateral ventricles before and after treatment with vehicle or RN 1734. The images are shown as coronal, sagittal, and horizontal plane images, and a 3D rendering of the lateral ventricles. Red and green are pseudocolors of the right and left lateral ventricles to provide additional definition of the fluid compartments.

Figure 3 illustrates examples of lateral ventricle CSF quantification using translational 3T MRI that uses the native contrast of the CSF by employing a T2W 3DSPACE imaging sequence (20) in WT, heterozygous (*Tmem67^+/–^*) and homozygous (*Tmem67^–/–^*) rat pups over an 8-day period from P7 to P15. Vehicle-treated animals are compared with those treated with the TRPV4 antagonist RN 1734 (P7–P14; i.p. daily, 4 mg/kg BW). MRIs were conducted on P7 and P15. The lateral ventricle volumes are indicated by red and green within horizontal, coronal, and sagittal views of the brain and as stand-alone 3D renderings (Figure 3).

**Figure 4:**
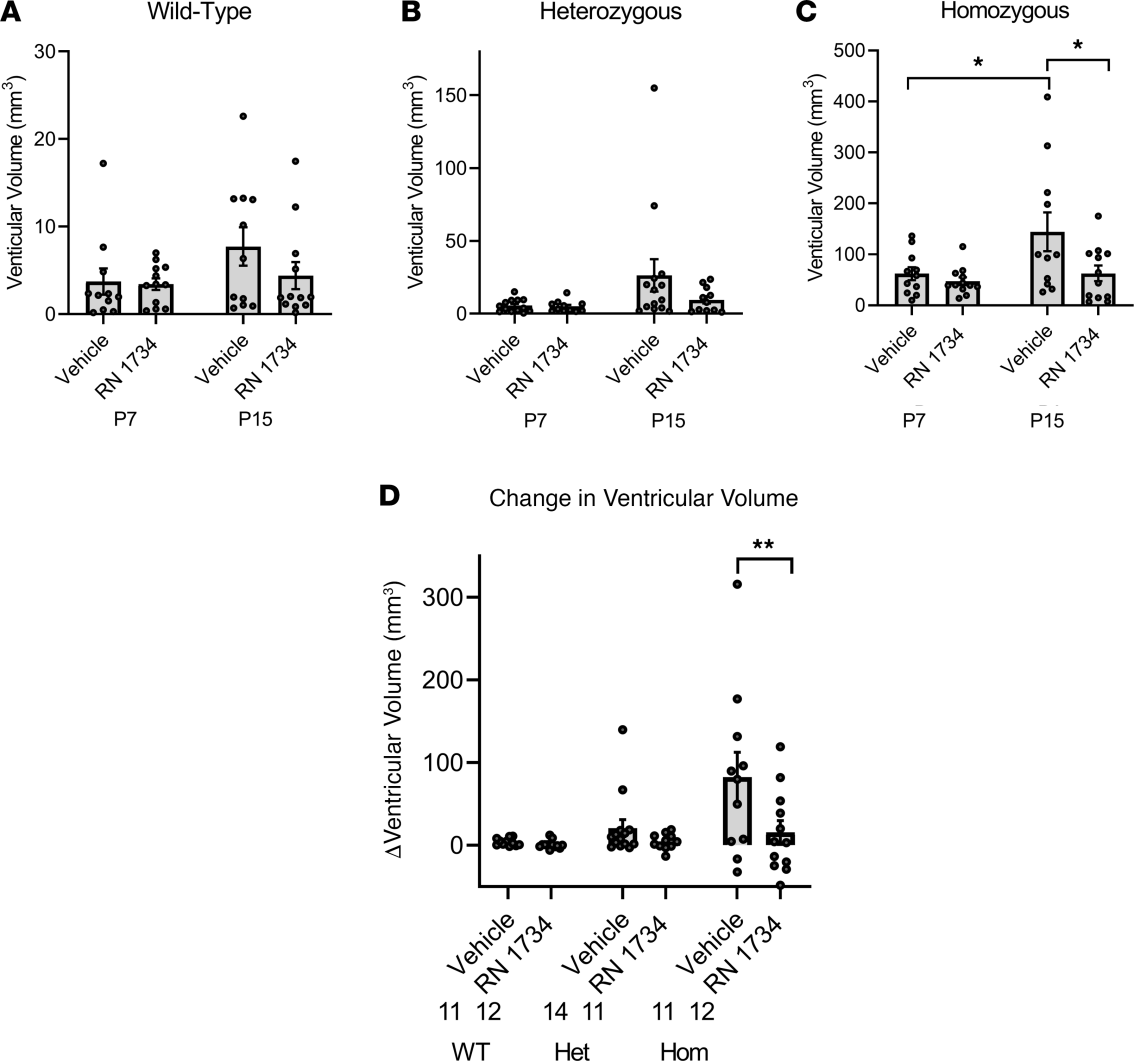
Quantitative measurements of amelioration of ventriculomegaly in hydrocephalic *Tmem67*^–/–^ rats by treatment with a TRPV4 antagonist, RN 1734. (**A–C**) WT (*Tmem67*^+/+^), heterozygous (*Tmem67*^+/–^), and homozygous/hydrocephalic (*Tmem67*^–/–^) ventricular volumes (mm^3^) at P7 and P15 in animals treated with either vehicle or RN 1734. (**D**) Change in ventricular volume (delta, Δ) from P7 to P15 for each genotype and treatment group. WT, vehicle (*n* = 11); WT, RN 1734 (*n* = 12); Het, vehicle (*n* = 14); Het, RN 1734 (*n* = 11); Hom, vehicle (*n* = 11); Hom, RN 1734 (*n* = 12). All data shown are the mean ± SEM for each group. Significance values were determined by 2-way ANOVA test in Prism using genotype and treatment as variables. Het, heterozygous; Hom, homozygous/hydrocephalic; vehicle = DMSO/saline daily i.p. injection; RN 1734, RN 1734, TRPV4 antagonist; 4 mg/kg BW i.p. daily injection.

Figure 4 provides the summarized ventricular volume data from the MRI study, comparing the lateral ventricle volumes of the vehicle-treated WT (Figure 4A), heterozygous (Figure 4B), and homozygous (Figure 4C) animals. Treatment with RN 1734 had no statistically significant effect on ventricular dilation in the WT or heterozygous animals. There was an approximately 20-fold increase in ventricular volumes in the homozygous animals compared with WT at both P7 (3.73 ± 1.50 versus 62.13 ± 12.58) and P15 (7.72 ± 2.19 versus 144.28 ± 38.06). By contrast, treatment with the antagonist completely inhibited the ventricular dilation of the homozygous animals between P7 and P15 (Figure 4C). Thus, the increased ventricular volume in the homozygous animals is mitigated by RN 1734 treatment. The composite results are illustrated in the bar graph showing the differences (Δ) in ventricular volumes between P7 and P15 for both genotypes and treatments (Figure 4D). The differences that were noted during disease development and treatment were independent of sex (Supplemental Figure 1; supplemental material available online with this article; https://doi.org/10.1172/jci.insight.137646DS1).

### Treatment with the TRPV4 antagonist has no adverse effects on body, kidney, or brain weight in WT or hydrocephalic animals.

**Figure 5:**
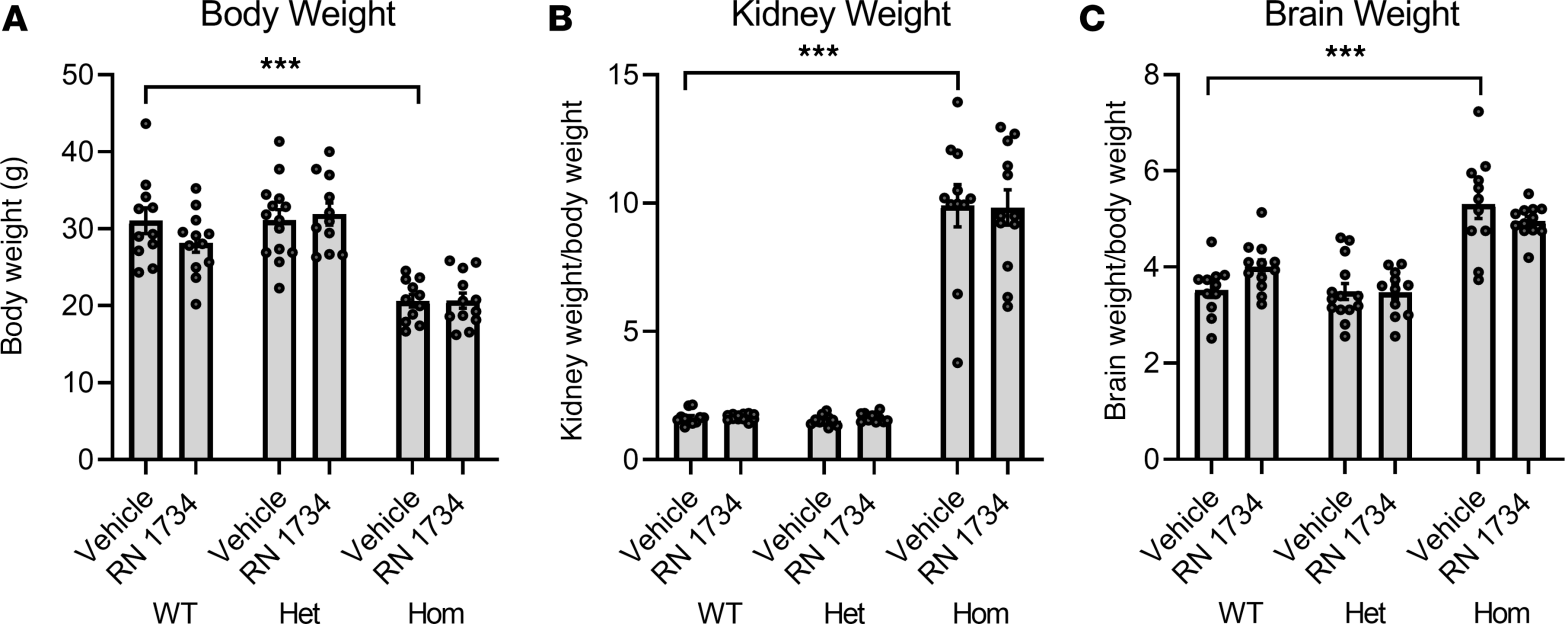
Effect on BW and organ size by treatment with TRPV4 antagonist RN 1734. (**A–C**) BWs (**A**), kidney weights (**B**) (combined left and right) expressed as a function of BW, and wet, intact brain weights (**C**) expressed as a function of BW collected at P15 for all groups. WT, vehicle (*n* = 11); WT, RN 1734 (*n* = 12); Het, vehicle (*n* = 14); Het, RN 1734 (*n* = 11); Hom, vehicle (*n* = 11); Hom, RN 1734 (*n* = 12). All data shown are the mean ± SEM for each group. Significance values were determined by 2-way ANOVA test in Prism using genotype and treatment as variables. Het, heterozygous; Hom, homozygous/hydrocephalic; vehicle = DMSO/saline daily i.p. injection; RN 1734, RN 1734, TRPV4 antagonist; 4 mg/kg BW i.p. daily injection

As a surrogate marker of whether the animals were feeding normally, we examined the effect of drug treatment on BW at the end of the study. While the hydrocephalic animals had a lower BW, the TRPV4 antagonist treatment had no effect on the BW of any of the genotypes (Figure 5A). As previously reported, the homozygous pups have severe polycystic kidney disease (16). In the current study, the homozygous animals had substantially enlarged kidneys, whether expressed as total kidney weight (data not shown) or as kidney weight as a percentage of overall BW (Figure 5B). Importantly, the kidney weight was not altered by TRPV4 antagonist treatment. Overall brain weights were not different across genotypes; however, when expressed as a percentage of BW, the brain weights in the homozygous animals were significantly higher (Figure 5C).

### Total amount of TRPV4 in CP is not changed by disease or drug treatment.

**Figure 6:**
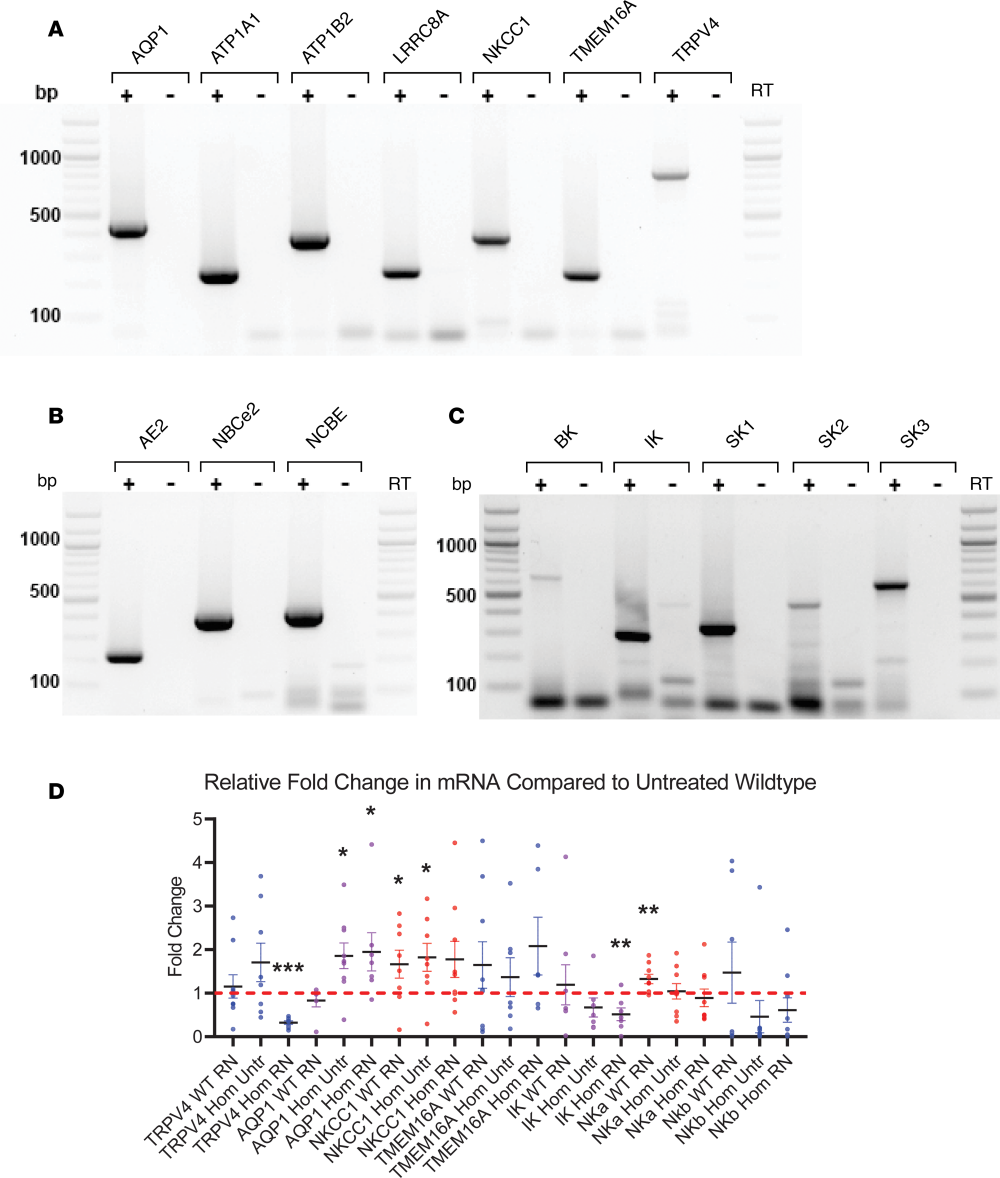
mRNA expression of water and electrolyte transporters and channels in native rat choroid plexus. (**A–C**) RT-PCR gels showing the presence of selected apical transporters (**A**), basolateral transporters (**B**), and potassium channels (**C**) in native rat choroid plexus tissue. (**D**) qPCR of WT and hydrocephalic (Hom) untreated (Untr) and RN 1734–treated (RN) choroid plexus (*n* = 3, each in triplicate) with TRPV4, AQP1 (WT RN, *n* = 2), NKCC1, TMEM16A, IK, Na^+^/K^+^ ATPase α (NKa) subunit and Na^+^/K^+^ ATPase β (NKb) subunit primers. RN treated choroid plexus tissue from homozygous animals showed a significant decrease (***P < 0.0001) in TRPV4 mRNA expression relative to untreated WT tissue. RN and Untr tissue from homozygous animals demonstrated significant (*P < 0.05) increases in AQP1 mRNA expression relative to untreated WT tissue. RN treated WT and Untr homozygous tissue also exhibited significant (*P < 0.05) increases in NKCC1 mRNA expression relative to untreated WT tissue. RN treated homozygous tissue had a significant (**P < 0.01) decrease in IK mRNA relative to untreated WT tissue. NKa mRNA increased significantly (**P < 0.01) in RN treated WT animals relative to untreated WT tissue. TMEM16A and NKb did not have any significant changes in mRNA regardless of genotype or treatment. Significance values were determined by unpaired *t* test calculated in Prism. AQP1, aquaporin 1; ATP1A1/B2, ATPase Na^+^/K^+^ Transporting Subunits α1/β2; LRRC8A, volume regulated anion channel; NKCC1, sodium, potassium, chloride cotransporter 1; TMEM16A, anoctamin-1 chloride channel; TRPV4, transient receptor potential vanilloid 4; AE2, acid exchanger 2; NBCe2, sodium bicarbonate cotransporter; NCBE, electrogenic sodium bicarbonate exchanger 1; BK, large conductance potassium channel; IK, intermediate conductance potassium channel; SK1/2/3, small conductance potassium channels 1/2/3. Primer information for can be found in Supplemental Table 1.

The rat is not the canonical model for studying electrolyte transport in the CP; therefore, we conducted PCR studies using freshly isolated CPs to confirm the presence of TRPV4 and other well-known transport proteins that have been demonstrated to be important in CP-mediated production of CSF (8). Figure 6 shows the reverse transcription PCR (RT-PCR) results indicating the presence of the apical membrane transporters, aquaporin 1 (AQP1), Na^+^K^+^ ATPase subunits α1 and β2, volume regulated ion channel, Na^+^K^+^2Cl^–^ cotransporter, TMEM16A chloride channel, and TRPV4, as well as the basolateral membrane transporters, anion exchanger 2, sodium bicarbonate cotransporter, and the electrogenic sodium bicarbonate exchanger. In a previous study, we documented the importance of Ca^2+^-activated potassium channels in TRPV4-mediated transepithelial ion flux (10). The rat CP expressed the intermediate conductance K^+^ channel (IK), as well as all 3 isoforms of the small conductance channels (SK1, SK2, and SK3).

Quantitative PCR (qPCR) of isolated CPs was used to determine potential changes in the gene expression of key signaling components, which might be expected to change as a function of disease development and/or treatment (Figure 6D). There was a statistically significant increase in AQP1 as a result of hydrocephalus (1.86 ± 0.29 relative to WT), and this was not reversed by antagonist treatment (1.95 ± 0.44). NKCC1, a triple cotransporter that has been implicated in CSF production, was increased by antagonist treatment in the WT animals (1.66 ± 0.04). The hydrocephalic state also caused an increase in expression of the NKCC1 (1.82 + 0.15), but this did not reach significance in the hydrocephalic, antagonist-treated animals. Compared with WT, TRPV4 gene expression did not change in response to antagonist treatment in the WT animals, nor was it statistically altered in the untreated hydrocephalic animals. Interestingly, there was a small but statistically significant decrease in TRPV4 mRNA in the treated, hydrocephalic animals. IK showed a trend toward lower levels in the homozygous animals, and this reached significance only in the antagonist treated homozygous group. Na^+^K^+^ ATPase α1 subunit increased moderately in the WT, treated animals but was unaffected in the hydrocephalic state. The TMEM16a chloride channel and the Na^+^K^+^ ATPase β2 subunit were unchanged by phenotype or treatment.

**Figure 7:**
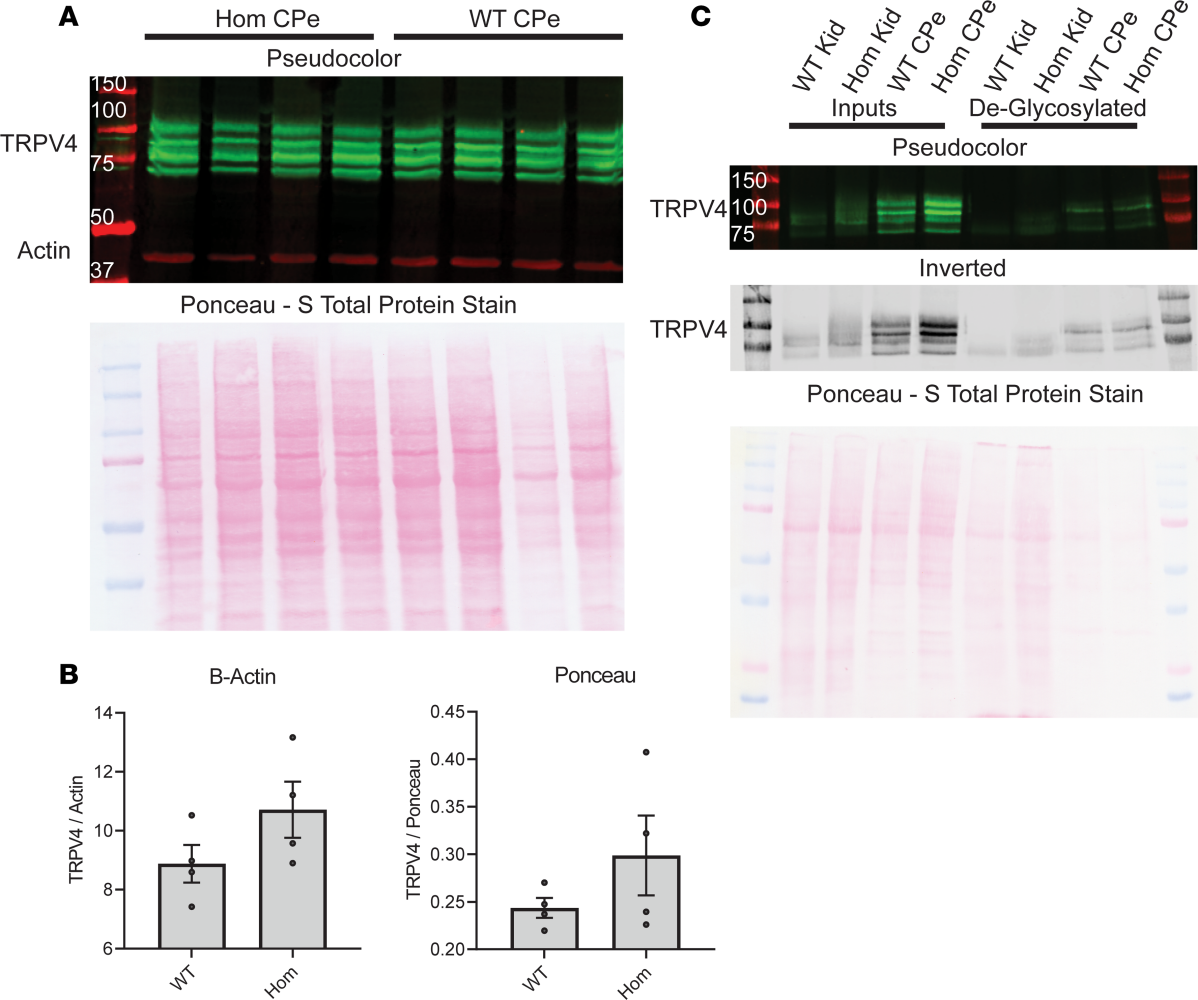
Expression of TRPV4 channel in native rat choroid plexus. (**A**) Immunoblotting of WT choroid plexus (WT CPe) and hydrocephalic choroid plexus (Hom CPe) for TRPV4 protein using β-actin and Ponceau S loading controls, showing no substantial change in protein expression of TRPV4 due to genotype. (**B**) Immunoblotting of WT kidney (WT Kid), hydrocephalic kidney (Hom Kid), WT choroid plexus (WT CPe), and hydrocephalic choroid plexus (Hom CPe) for TRPV4. (**C**) Deglycosylation of TRPV4 with PNGaseF enzyme with matched untreated inputs demonstrating that there are 2 isoforms of TRPV4, both of which are glycosylated in the choroid plexus. There is only 1 isoform of TRPV4 in the kidney, but it is also glycosylated. Significance values were determined by paired *t* test in Prism between experimental groups, and no significant differences were found.

Given the importance of TRPV4 as a hub protein, the qPCR results were confirmed by Western blotting of tissue from freshly isolated CPs (Figure 7). Western blots of total protein isolated from CP tissue indicate that there is no significant change in TRPV4 protein expression between untreated WT and homozygous animals as normalized to either β-actin or Ponceau S loading controls (Figure 7, A and B). The observed number of distinct bands recognized by the anti-TRPV4 antibody is unusual. Therefore, additional blotting and specificity studies were performed. Antibody specificity was determined by immunoprecipitation and subsequent detection by MS/MS at the Indiana University Proteomics Core. The MS/MS analysis confirmed that TRPV4 was immunoprecipitated by the antibody (data not shown). Additionally, immunoprecipitation with an IgG control was also used as a specificity determinant (data not shown).

The presence of 4 distinct TRPV4 bands in the CP as opposed to 2 bands in kidney tissue from the same animals (Figure 7C) indicated the presence of multiple isoforms and/or posttranslational modification of the protein. To determine if the protein was glycosylated, samples were treated with PNGaseF to deglycosylate an aliquot of the samples before running on a gel in tandem with the untreated samples. The deglycosylation resulted in a single protein band in the samples obtained from the kidney and 2 distinct bands in the proteins of the CP (Figure 7C). To our knowledge, this is the first demonstration of glycosylated isoforms of TRPV4 in the CP that are distinct from the isoform found in renal tissue. The glycosylation patterns are interesting and indicate that similar levels of expression of glycosylated and nonglycosylated forms of the transporter exist in the CP.

The finding that the amount of TRPV4 did not change suggests that the mechanism of the TRPV4 effect on CSF production could be mediated via a change in cell surface expression or an activation of channels already present on the cell membrane.

### Mechanism of action of TRPV4 in CP.

TRPV4 is a nonspecific cation channel whose activation is usually associated with the influx of Ca^2+^ (14, 21). However, the nonselective nature of the channel allows for the transport of other cations, notably Na^+^ (22). The changes in intracellular ion concentrations secondarily alter other channels and transporters leading to the transepithelial flux of both electrolytes and water (10, 11, 23).

**Figure 8:**
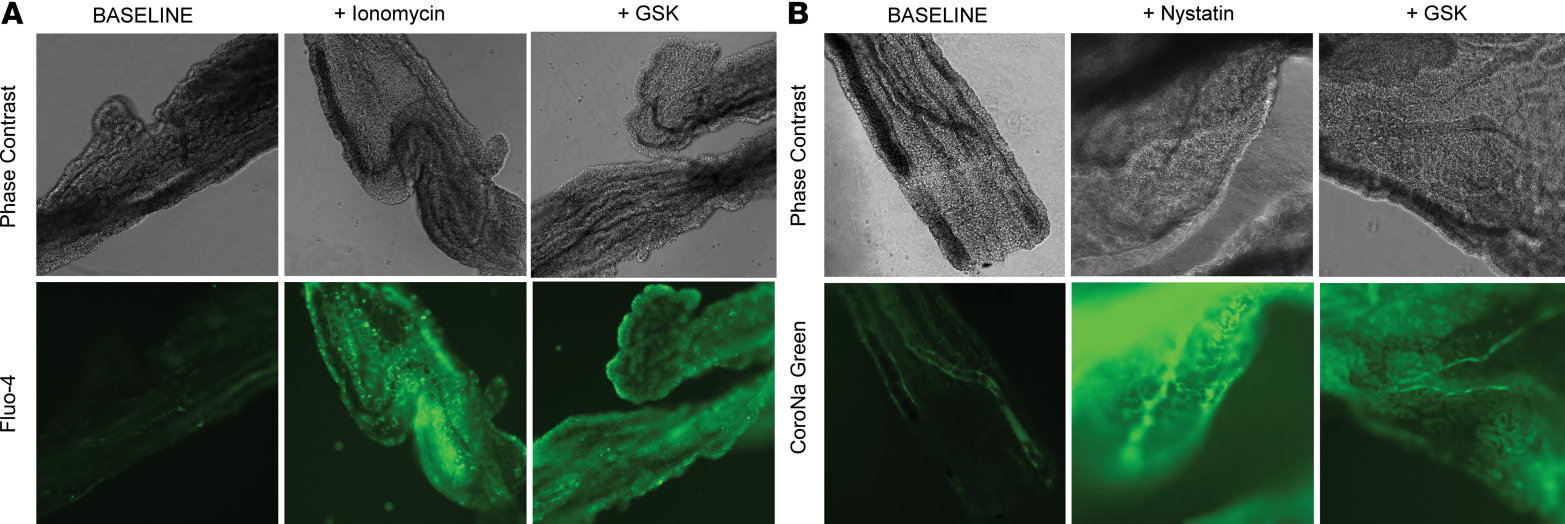
TRPV4 activation elicits functional sodium and calcium influx into ex vivo rat choroid plexus. (**A**) Ex vivo choroid plexus was incubated in Fluo-4 calcium indicator dye to study calcium influx into the epithelial cells. Ionomycin (100 μM) was used as a positive control for calcium influx, and GSK1016790A (3 nM) was used to agonize TRPV4. TRPV4 activation generated a qualitative increase in fluorescent signal, consistent with allowing calcium influx into the cells. (**B**) Ex vivo choroid plexus was incubated in CoroNa Green Sodium indicator dye to study sodium influx into the epithelial cells. Nystatin (100 μM) was used as a positive control for sodium influx, and GSK1016790A (3 nM) was used to agonize TRPV4. TRPV4 activation generated a qualitative increase in fluorescent signal, consistent with allowing sodium influx into the cells. TRPV4, transient receptor potential vanilloid 4; GSK, GSK1016790A. Original magnification, 40×.

Figure 8A shows the effect of TRPV4 activation on the influx of Ca^2+^ in freshly isolated CP from the rat model. Activation of TRPV4 with an agonist caused an immediate increase in intracellular Ca^2+^ that was similar to the positive control using the Ca^2+^ ionophore, ionomycin, to induce Ca^2+^ influx. Interestingly, using a sodium indicator showed that channel activation simultaneously stimulated the influx of extracellular Na^+^ in CP, which is similar to that observed with the Na^+^ ionophore nystatin (Figure 8B). These substantial and immediate changes in the intracellular ionic concentrations will undoubtedly cause compensatory activation of other channels and intracellular signaling pathways, leading to transepithelial electrolyte and fluid flux such as that observed by multiple investigators, including ourselves (10, 11, 23, 24).

## Discussion

Although investigators have been attempting to develop nonsurgical treatments for hydrocephalus for over 60 years, successes in preclinical animal studies have not translated well into clinical trials (25). In part, this may be due to preclinical models that do not accurately recapitulate the human condition initiation or progression. For example, one of the most common models of induced pediatric hydrocephalus involves injections of kaolin into the cisterna magna or the cerebral ventricles. This generally produces an obstructive form of hydrocephalus, but it has been well recognized that kaolin is a caustic chemical that causes denudation of the epithelial cells lining the CP and ventricular wall (26). Therefore, this treatment destroys the barrier epithelial cells that one wishes to study with regard to the control of CSF production and whose dysregulation may contribute to the pathological changes that lead to hydrocephalus. The development of models that reflect the pathological and compensatory changes that occur during hydrocephalic development are necessary before preclinical studies will be translatable to patients. Our current studies were performed in a genetic rat model, which is more physiologically relevant to the clinical condition.

Commonalities in the communicating forms of hydrocephalus include ventricular enlargement coupled with an imbalance in fluid-electrolyte homeostasis. This can be due to overproduction or underabsorption of CSF. Alternatively, there may be a change in the ionic composition of the CSF, leading to fluid accumulation via osmotic forces. Regardless of the initiating cause of the disease, treating the changes in fluid-electrolyte balance may provide an effective treatment. Therefore, good targets for drug development are the electrolyte transport proteins of the epithelial cells of the CP that are responsible for the production and unique composition of the CSF, particularly those that are likely to be activated by pathophysiological changes such as inflammatory mediators or alterations in pressure or fluid flux.

In the Tmem67^–/–^ genetic model of communicating hydrocephalus, we have shown that 2 different TRPV4 antagonists inhibit the development of ventriculomegaly. These data are in agreement with in vitro studies in cultured CP epithelia demonstrating that TRPV4 agonists stimulate a multiphasic transepithelial ion flux that can be inhibited by either of the 2 antagonists used in the current study (10–12). The mechanism of action and the role of TRPV4 in the development of hydrocephalus do not appear to be dependent on increased expression of the channel protein, suggesting that the mechanism is, rather, a change in cell surface expression and/or activation. This finding is also in agreement with the cell culture data where activation of TRPV4 changes epithelial permeability and transepithelial ion transport within minutes (10, 11), not within the hours required for new protein synthesis. These data are consistent with the hypothesis that TRPV4 is activated by the pathophysiological changes that initiate the process of hydrocephalus. TRPV4 has been shown to be activated by multiple inputs from chemical (arachidonic acid metabolites and cytokines) to physical changes (pressure and osmotic changes) (14, 21). All of these are applicable within the myriad causes of hydrocephalus. In this regard, it should be noted that the TRPV4 antagonists do not appear to alter ventricular volumes in normal animals and are, therefore, applicable primarily in the hydrocephalic state. We speculate that our findings of TRPV4 antagonist–mediated inhibition of the development of hydrocephalus will be applicable to most forms of the disease and could be used on an as-needed basis.

Other key transport elements show only moderate changes in expression due to hydrocephalus. Treatment with the TRPV4 antagonist does not restore the AQP1 to control levels, indicating an independence in cell signaling between AQP1 and TRPV4. Interestingly, blocking TRPV4 causes an increase in NKCC1 in the WT animals, potentially as a compensatory change in electrolyte flux across the tissue. This increase in the triple cotransporter is also seen in response to the hydrocephalic state, but the increase, while trending higher, was not statistically maintained during antagonist treatment. These results are preliminary but indicate a complex regulation of water/electrolyte balance that deserves further study.

Pharmacologically altering the production of CSF is a promising approach with the potential to revolutionize treatment and spare patients the cost and morbidity related to surgery. TRPV4 inhibitors are in clinical trials for a variety of other indications. GlaxoSmithKline (GSK) has completed several short-term clinical trials using GSK2798745, an orally bioavailable TRPV4 channel blocker (https://clinicaltrials.gov.). In a 7-day trial in patients with congestive heart failure, the primary measure of changes in pulmonary gas transfer and respiration was not statistically analyzed because of small sample size (*n* = 11). Importantly, there was neither mortality nor any serious adverse clinical effects attributable to the drug. Subsequently, GSK conducted additional human studies for chronic cough and alveolar barrier disruption in segmental LPS challenge. In both of these studies, the investigators declined to analyze outcomes but also reported no mortality and no serious adverse effects in any of the volunteers. Finally, a recent report describes the first-in-human study to evaluate safety, tolerability, pharmacodynamics and pharmacokinetics of GSK2798745 in healthy subjects and stable heart failure patients. Five cohorts contained a total of 60 participants who were treated with the TRPV4 antagonist (27). Again, the drug was well tolerated, and there was no mortality and were no serious adverse clinical effects. These human studies suggest that inhibiting TRPV4 will have minimal side effects, at least in the short-term. This was presaged by animal studies that showed that *Trpv4*-null mice have a normal appearance, growth, and reproductive capacity and only develop osmotic abnormalities when placed under severe osmotic stress (28). The published studies combined with our current data suggest that inhibition of TRPV4 may be a safe and effective treatment for some forms of hydrocephalus.

## Methods

### Study design.

The objective of this research was to test the effect of TRPV4 antagonists on ventricular volume in a genetic rat model of hydrocephalus, *Tmem67*^–/–^. The rat colony was provided by Vincent Gattone (Department of Anatomy and Cell Biology, Indiana University School of Medicine, Indianapolis, Indiana, USA). Based on preliminary head dimension data, it was hypothesized that the TRPV4 antagonists would reduce ventricular volume in the hydrocephalic rat pups. These were controlled laboratory experiments, and details of the treatment and measurement methods can be found in the Results sections. Animals in these studies were randomly chosen for either vehicle or drug treatment. Head dimension measurement series were conducted by the same observer who was blinded to the genotype of the animals. In subsequent studies, the MRI imaging technicians and analysts were blinded to both genotype and treatment of the animals. Animals were excluded from the study only by death before the final MRI (5 animals). Heterozygous *Tmem67^+/–^* animals were bred to generate WT (*Tmem67^+/+^*), heterozygous (*Tmem67^+/–^*), and homozygous (*Tmem67^–/–^*) pups to be used in treatment studies.

### Genotyping.

Tail snip samples were collected from pups before P3. DNA was extracted using the reagents and protocol of the QIAGEN QIAamp DNA Mini Kit 250 (catalog 51306). After extraction, PCR was performed using 0.25 μL mixture of primers 5′ AGA AAA GTT CTT CAC TGG TTG ACA 3′ (forward) and 5′ CAT CAT CAT CCC TGG TTC CTG 3′ (reverse), 6.25 μL Quanta Bio’s AccuStart GelTrack PCR SuperMix (catalog 95136), and 4 μL DDI H_2_O. The reaction was run at 95°C for 2 minutes, followed by 35 cycles of 95°C for 15 seconds, 60°C for 30 seconds, and 72°C for 1 minute, followed by 72°C for 1 minute. After completion of PCR, excess nucleotides were removed using 4 μL of applied biosystems ExoSAP-IT Express PCR Product Cleanup (catalog 750011). The reaction was run at 37°C for 1 minute, followed by 37°C for 14 minutes, and ending with 80°C for 15 minutes. Samples were sent to Eton Biosciences with the forward primer for sequencing to analyze the C1186T loci of exon 12 of the *Tmem67* gene.

### Head measurements.

Before and after drug treatment (days listed in figure legends) both vertical (palate to cranial cap) and horizontal (biparietal) head measurements were taken using calipers. In the experimental series using the TRPV4 antagonists HC067047 and RN1743, the control group represented both WT and heterozygous animals. In this early experimental set, the complex genotyping had not yet been established, and the animals were characterized as homozygous or hydrocephalic, if their kidneys were enlarged. Heterozygous animals do not have enlarged kidneys. We feel these head measurements are valid because the extent of the hydrocephalus is not sufficient in the heterozygous animals to alter the head dimensions.

### Treatment protocol for MRI study.

Pups were randomly chosen for either vehicle or drug treatment. Pups underwent preliminary MRI on P7 and then were treated daily with an i.p. injection of either RN 1734 (Tocris, 3746), 4 mg/kg BW, or equal volume 100% DMSO vehicle. On P15, pups underwent final MRI; they were then sacrificed, and tissue was procured.

### Anatomical MRIs.

On P7 and P15, rat pups were briefly removed from their litter and induced with 5% isoflurane (balance medical oxygen), and anesthesia was maintained with 1%–2% isoflurane (balance medical oxygen). High-resolution T2-weighted (T2W) MRI images were acquired using a 3T Siemens Prisma clinical MRI scanner outfitted with a dedicated 4-channel rat head coil and bed system (RapidMR). Images were acquired using a SPACE3D sequence with the following acquisition parameters: (TA:,5.5 minutes; TR, 2080ms; TE, 162ms; ETL, 57; FS, On; Ave, 2; Excitation Flip Angle, 150; Norm Filter, On; Restore Magnetization, On; Slice Thickness, 0.2 mm: Matrix, 171 × 192; FOV, 35 × 35 mm) yielding 0.18 × 0.18 × 0.2 mm resolution images. Volumes of interest (VOI) of lateral ventricles were determined from threshold-based image segmentation of native cerebrospinal fluid contrast, where images were quantified for lateral ventricular volumes using Analyze 12.0 (AnalyzeDirect). In all cases, study personnel were blinded to genotype and treatment during acquisition and analysis.

### RT-PCR.

Animals were euthanized via CO_2_ exposure followed by rapid decapitation, and brains were harvested. Lateral ventricle CPs were resected and flash frozen. Total cell RNA was collected using the Monarch Total RNA Miniprep Kit (New England Biolabs, T2010S) using the manufacturer’s directions for mammalian tissue. RNA concentration was measured using an ND2000 NanoDrop (Thermo Fisher Scientific). Approximately 100 ng of total RNA was reverse transcribed into cDNA using the Monarch LunaScript RT SuperMix Kit (New England Biolabs, E3010L), along with corresponding negative RT (–RT) cDNA control and a template control not containing RNA (NTC), according to the manufacturer’s directions. Rattus norvegicus exon mRNA sequences for each gene were obtained using Ensembl, and primer pairs for each were designed using Primer3Plus. Approximately 500 ng of template cDNA was combined with the forward and reverse primers (IDT), as well as GoTaq Green Master Mix (Promega Corporation, M7122). Reactions were run as a gradient to determine optimum annealing temperature for each primer pair, and products were separated on a 1.5% agarose gel with ethidium bromide. Flanking 100 bp ladders were used as molecular weight markers, and gels were imaged using a ChemiDoc XRS imager (Bio-Rad). Single band amplicons of the correct molecular weight were sequenced (Eton Biosciences), and the correct products were validated using NCBI and Ensembl BLAST.

### qPCR.

CP RNA was collected and transcribed as described for RT-PCR. The cDNA was diluted with nuclease-free water (New England Biolabs). All samples were run in triplicate. qPCR was performed using a LightCycler 480 Instrument II real-time PCR system (Roche LifeScience), using LightCycler 480 SYBR Green I Master Mix (Roche LifeScience, 04707516001). qPCR cycle conditions were 95°C for 5 minutes, followed by 45 cycles of 95°C for 10 seconds, 60°C for 10 seconds, and 72°C for 10 seconds. Data are displayed as relative fold change in expression using the 2^–ΔΔCT^ method (29), relative to the calibrator housekeeping genes GAPDH and Rps18. Data are shown as fold change in each compound of interest in treated WT and treated and untreated homozygous animals relative to the normalized control (untreated WT) animals. Primers were validated by sequencing. Supplemental Table 1 contains primer information.

### Western immunoblots.

Animals were anesthetized and euthanized with rapid decapitation. The brain was harvested, and the lateral ventricle and third ventricle CPs were harvested and flash frozen. Samples were solubilized with low ionic lysis buffer (0.05% EDTA, 0.5% Triton X-100 [Thermo Fisher Scientific], 0.1% Tris-HCl, 0.1% DTT, 0.1% protease inhibitor cocktail [BioMake], 0.5% sodium orthovanadate, 0.5% sodium fluoride, 0.5% β-mercaptoethanol, 0.5% sodium pyrophosphate [all from MilliporeSigma]) with vigorous pipetting followed by brief pulse sonication. Sample buffer (4×) was added, and samples were heated at 70°C. The resulting solubilized samples were run on a 10% gel at 200V for 45 minutes. Proteins were transferred to nitrocellulose membranes and were Ponceau S stained for total protein. Subsequently, membranes were blocked with 5% nonfat milk (Carnation) in TBS-T and probed with rabbit anti-TRPV4 antibody (Invitrogen, PA5-77319) or a β-actin antibody (Proteintech, 60008-1-Ig) overnight at 4°C. The following day, the membranes were washed and blocked with 5% nonfat milk in TBS-T and then probed with secondary Alexa Fluor anti-mouse 690 nm or anti-rabbit 790 nm (Jackson ImmunoResearch; 715-625-151 and 711-655-152, respectively) antibodies. Membranes were washed with TBS and visualized on a LICOR Odyssey machine. Deglycosylation was performed using PNGaseF enzyme recombinant (New England Biolabs, P0708S) according to the standard protocol provided by the company. Band intensities were quantified using the LICOR software and normalized to both β-actin and Ponceau S total protein stain as quantified using ImageJ software (NIH).

### Cation dyes.

Animals were briefly anesthetized and euthanized with rapid decapitation. The brain was removed, and lateral ventricle and third ventricle CPs were harvested and placed in warm DMEM high glucose without phenol red. Suspended tissues were allowed to equilibrate at 37°C for 30 minutes. Subsequently, tissues were incubated in either Fluo-4 AM dye (Invitrogen, F14201) or CoroNa Green AM dye (Invitrogen, C36676) for 30 minutes at 37°C according to Invitrogen protocols. Tissues were washed twice with DMEM high glucose without phenol red. The TRPV4 agonist, ionomycin, or nystatin were added, and the tissues were imaged on a Keyence BZ-X800 epifluorescence scope. Image processing was done in the Keyence BZ-X800 Analyzer software, and figures were arranged in PowerPoint.

### Statistics.

Graphs were produced in Prism software; figures were arranged in Prism, PowerPoint, and Adobe Illustrator; and statistical analyses were performed using SigmaPlot (version 14, Systat Software Inc.). Power analysis was performed routinely until the power of the study exceeded 0.50 (50%). Data were tested for normality using the Shapiro-Wilk test, and equal variance was tested using the Brown-Forsythe test. Normally distributed data were analyzed using 2-tailed Student’s *t* test or 2-way ANOVA when comparing 2 or 3 groups, respectively. Nonnormally distributed data were analyzed using the nonparametric Mann-Whitney *U* and the Holm-Šidák method post hoc tests, which compare 2 and 3 data groups, respectively. Data were expressed as mean ± SEM, and *P* < 0.05 was considered significant.

### Study approval.

Animal experiments were performed under approved protocols from the IACUC of Indiana University, Purdue University, Indianapolis.

## Author contributions

AEH and HMS contributed equally to the project and, thus, serve as co–first authors. They are listed in alphabetical order. AEH, HMS, and BLBY drafted the manuscript and prepared figures. AEH helped with animal studies and the development of genotyping, performed immunoblotting and ex vivo CP imaging, and generated figures for the manuscript. HMS designed and conducted animal studies and genotyping, and generated figures for the manuscript. PRT designed and directed the MRI studies. DCP and MMR designed and conducted PCR and qPCR studies. JWS was involved in the early animal studies and contributed to development of genotyping. DF served as a clinical consultation in these studies. BLBY designed experiments, conducted data analyses, verified all data in the manuscript for accuracy, and finalized the manuscript. All authors have read and approved the submitted version of the manuscript.

## Supplementary Material

Supplemental data

## Version 1 09/17/2020

Electronic publication
