# Supplementary material for: TRPV4 antagonists ameliorate ventriculomegaly in a rat model of hydrocephalus
Source: JCI Insight. 2020 Sep 17;5(18):e137646. doi: 10.1172/jci.insight.137646 (PMC7526552; doi:10.1172/jci.insight.137646)
Supplement: Supplemental data [file jciinsight-5-137646-s009.pdf]

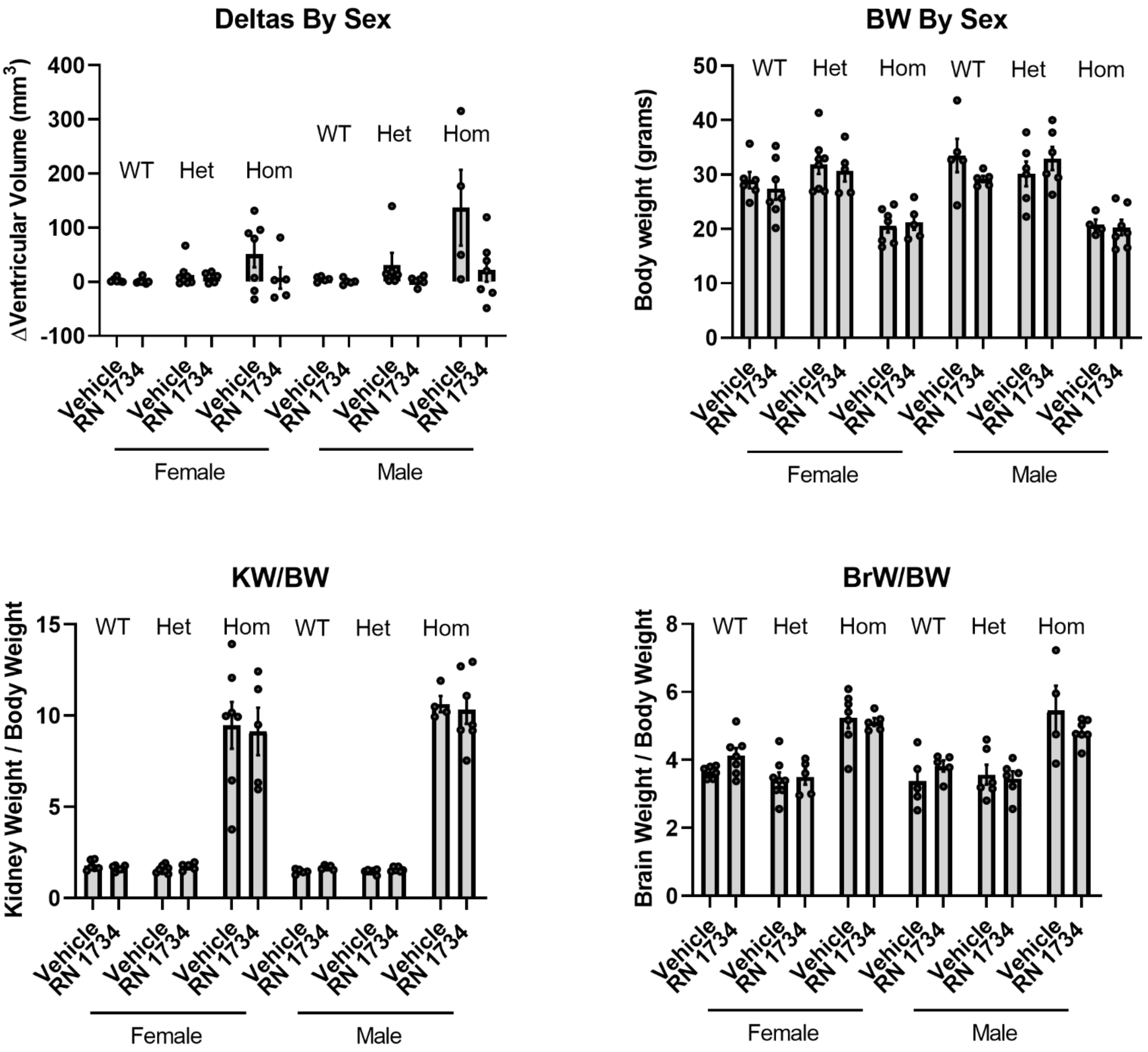

Supplemental Figure 1: Quantitative measurements of Tmem67$^{+/-}$ and Tmem67$^{-/-}$ rats treated with RN 1734 with sex differences considered.

(A) Change in ventricular volume from P7 to P15 for rats of all genotypes after treatment with either vehicle or RN 1734 with male and female data separated. (B) Body weight at P15 with male and female data separated. (C) Kidney weights, expressed as a function of body weight, with male and female data separated. (D) Brain weights, expressed as a function of body weight with male and female data separated. No significant differences were found when considering sex as a variable. All data shown are the mean ± sem for each group. Significance values determined by two-way ANCOVA test in Prism using genotype, treatment, and sex as variables. P15 = postnatal day 15; mm = millimeters; Veh = DMSO/Saline daily i.p. injection; RN = RN 1734, TRPV4 antagonist, 4 mg/kg BW i.p. daily injection.

Supplemental Table 1

Rattus Norvegicus gene	Protein	Primer Sequences 5' - 3'		Product Size (bp)
		Forward Primer	Reverse Primer	
SLC4A2	AE2	AGACAGCCCGGTGGATAAAATT	CTGTCCTCTGCTTTGATCTGGT	223
AQP1	AQP1	CCTCCGGGCTGTCATGTATATC	ACCTTCATGCGGTCTGTAAAGT	459
ATP1A1	ATP1A1	AGGAATTCTGTCTTCCAGCAGG	GTAGGTTTCCTTCTCCACCCAG	246
ATP1B2	ATP1B2	AAAGAATGATGTCTGCCGTCCT	TACATTCACCTCCACATTGGGG	406
KCNMA1	BK	CAGGTGGAGTTTTATCAGGGCT	ATCATGACAGGCCTTGCAGTAA	675
KCNN4	IK	CAACTTCCTTGGAGCAATGTGG	CAGGGTGACGATCCTTTTCTCA	511
LRRC8A	LRRC8A	CAACCGCTACATCGTCATTGAC	AGGTTGTGGAGGCTAAAGATGG	255
SLC4A5	NBCe2	AGGGAGTAATGGAGAGCTTCCT	ACTCGCATTTGTAGGTGGTGAT	390
SLC4A10	NCBE	CCATATTAGGCAGTACGGGACC	CTCTGACACAGTTAGGTTCCCC	416
SLC12A2	NKCC1	TCGATTGTCATGGATTGTGGGT	GGCCAGAAGAAGAATCACCAGA	418
KCNN1	SK1	CCAAGGAGTCTCTGTGCTCATT	ATGGACAGAAGCACATCCACAT	298
KCNN2	SK2	CAACTTCCTTGGAGCAATGTGG	CAGGGTGACGATCCTTTTCTCA	511
KCNN3	SK3	TCTGTCCATCCCCATGTTCTTG	CCCCTCAGTTGGTGAATAGCTT	621
ANO1	TMEM16A	ATCTCCAAAGACTTCTGGGCT	TGTGGGACTGTGGTTGTTACAA	261
TRPV4	TRPV4	CTCGCCCTTCAGAGACATCTAC	TGATAGTAGGCTGTGAGGGTGA	792
GAPDH	GAPDH	CCTGGAGAAACCTGCCAAGTAT	GACAACCTGGTCCTCAGTGTAG	103
RPS18	RPS18	CATGTGGTGTTGAGGAAAGCAG	TATTGTCGTGGGTTCTGCATGA	107

Supplemental Table 1: Primer Pairs used for RT-PCR and qRT-PCR.

Three redundant primer sets were generated and tested for each gene. Primers included in this table were utilized in Figure 6. Single band amplicon products were sequenced to verify correct gene amplification product. AQP1 = Aquaporin 1; ATP1A1/B2 = ATPase Na+/K+ Transporting Subunits Alpha 1/Beta 2; LRRC8A = Volume Regulated Anion Channel; NKCC1 = Sodium, potassium, two chloride cotransporter 1; TMEM16A = Anoctamin-1; TRPV4 = Transient receptor potential vanilloid 4; AE2 = Acid Exchanger 2; NBCe2 = Sodium bicarbonate cotransporter; NCBE = Electrogenic sodium bicarbonate exchanger 1; BK = Large conductance potassium channel; IK = Intermediate conductance potassium channel; SK1/2/3 = Small conductance potassium channels 1/2/3. GAPDH = Glyceraldehyde 3-phosphate dehydrogenase; RPS18 = Ribosomal protein subunit 18
